# Physiological Effects of Psychological Interventions Among Persons with Financial Stress: A Systematic Review, Meta-analysis, and Introduction to Psychophysiological Economics

**DOI:** 10.1007/s10484-024-09658-x

**Published:** 2024-09-27

**Authors:** Paul Lehrer, Lilly Derby, Jacqueline Smith Caswell, John Grable, Robert Hanlon

**Affiliations:** 1 Rutgers Robert Wood Johnson Medical School and Centerline Institute, New Brunswick, NJ USA; 2 The State University of New Jersey, New Brunswick, NJ USA; 3 University of Georgia, Athens, GA USA; 4 Centerline Institute, Easton, PA USA

**Keywords:** Financial problems, Stress management, Psychophysiological economics, Psychotherapy, Cognitive behavior therapy, Relaxation therapy, Inflammatory cytokines

## Abstract

It is known that economic problems can cause psychological stress, and that psychological stress causes physiological changes often linked to disease. Here we report a systematic review and meta-analysis of studies on physiological effects of psychological treatment for individuals with economic problems. Of 5071 papers in our initial PsycInfo search, we identified 16 papers on physiological effects for psychological treatment of the economically stressed. We found 11 controlled studies, among which we found a small to moderate significant effect size, Hedges’ *g* = 0.319, *p* < 0.001. The largest effect sizes were found for heart rate variability and measures of inflammation, and the smallest for measures involving cortisol. The studies were all on chronically poor populations, thus restricting generalization to other financially stressed populations such as students, athletes in training, and those stressed by relative deprivation compared with neighbors or other reference groups. None of the studies examined effects of these psychophysiological changes on disease susceptibility, and none included elements of financial planning. The nascent field of financial psychophysiology calls for more research in these areas. Even so, results suggest that financially stressed people can benefit physiologically from psychological stress management methods.

## Introduction

Stress has many causes and many manifestations. The stress response may include various characteristics: cognitive, behavioral, and physiological. Cognitive components include thought patterns that can spiral minor stressors into major ones. Behavioral components include avoidance of activities, performance decrements, and problems in interpersonal relations. Physiological components can include the various physical manifestations of the stress response, as well as predisposition to stress-related disease. These three dimensions of stress do not always occur together (Lang, 1968) and each may require a specific treatment component (Martin, 1991). This report focuses on the physiological component, with its possible link to stress-related disease.

Physiological variability is not necessarily problematic and can be adaptive. Variability, by itself, is actually an index of health, defined by McEwen (1998) and his colleagues as “allostasis,” or stability by variability. The body responds to almost all tasks and stimuli, allowing the individual to respond appropriately to large and small events. Also, various endogenous sources of variability reflect the operation of control mechanisms that maintain homeostasis after event-related changes (Lehrer & Eddie, 2013). However, when environmental demands are severe or prolonged, the body is stressed, and allostatic overload can occur where the body’s resources are taxed to the point where health, performance, and sense of well-being are impaired. Allostatic overload can produce a variety of physiological effects and symptoms (Kusnecov et al., 2021; Lehrer, 2021). It is the “stress” response.

The stress response can include increased sympathetic arousal, ventilation, and inflammation. These can provide more oxygen and sugar to the muscles for the “fight-flight” response and protection against infection from injury. Although this response may be adaptive for coping with physical stressors, perhaps explaining their evolutionary role, in modern life stress is more often are triggered by social demands, where the need for physical mobilization or infection control are not needed. This can cause imbalance in the body’s homeostatic network and can lead to stress symptoms.

Prominent among social stressors are those caused by economic problems. The resulting physiological reactions are the subject matter for the new field of psychophysiological economics (Grable, 2013). Financial stress occurs in the absence of a sense of financial well-being, which includes dimensions that are both objective (insufficient money) and subjective (one’s interpretation of financial condition as being poor) (Friedline et al., 2021; Sorgente et al., 2022). It often occurs when a household is unable to meet ongoing financial obligations, and has been operationalized as the physical or mental health symptoms that arise from having difficulty meeting basic needs, difficulty paying bills, and lack of money left over at the end of a month (Valentino et al., 2014). Greater income and wealth therefore are associated with measures of lower financial stress (Romo 2014; Valentino et al. 2014). In their development of a validated scale to measure financial stress Northern et al. (2010) define the construct as “an inability to meet one's economic responsibilities … influenced by psychological factors such as attitudes, beliefs, and cognitive appraisals of demands and available resources.” In their systematic review, Guan et al. (2022) used both objective and subjective definitions of financial stress.

There is ample evidence that financial difficulties are associated with a more general sense of psychological stress and impaired well-being (Algren et al., 2018; Yan et al., 2023). In their periodic survey of stress in America, the American Psychological Association regularly identifies financial problems as major sources of stress symptoms. In 2022 83% of respondents reported inflation as a stressor, 57% reported not having enough money, and 43% reported the inability to save enough money for future needs (American Psychological Association, 2022).

Economic stress can occur over extended periods of life, and chronic stress has been linked to allostatic overload and to a variety of diseases (Guidi et al., 2021). Stress-related psychophysiological markers have been found for dermatological disorders (Bewley et al., 2021), heart disease (Cundiff & Smith, 2017), hypertension (Brugge, 2022; Sweet, 2021), back pain (Ochsmann et al., 2009), asthma (Lehrer & Moritz, 2023), gastrointestinal problems (Jepson, 2008; Overmier & Murison, 2013) and general disease vulnerability (Bellingrath & Kudielka, 2017; Dar et al., 2019; Lovallo, 2016; Mariotti, 2015; McGrady & Moss, 2013). Chronic stress also contributes to various emotional disorders (Berretz et al., 2020; Linden & Stuart, 2020; Staufenbiel et al., 2014).

A link between economic hardship and disease is well established (Arega et al., 2022; Pond, 1961; Singh & Singh, 2008; Semyonov et al., 2013; Turner et al., 2020), although the link can be mediated by individual differences in stress reactivity (Zankert et al., 2019) and access to healthcare (Agency for Healthcare Research and Quality (US), 2021). Economic stress also is associated with obesity and other indices of poor health (Bruening et al., 2018) and tends to be associated with emotional difficulties (Boschert et al., 2020; Financial Health Network, 2023), particularly anxiety disorders (Jovanovic & Norrholm, 2016; Ramakers et al., 2003), depression (Jimenez et al., 2021; Luo et al., 2009), and marital instability (Hill et al., 2017).

The association between emotional problems and financial difficulties may be bidirectional and create a vicious cycle of both conditions. Not only do financial problems produce stress, but the experience of psychological trauma may impact a person’s ability to make financial decisions (Falconier, 2015; Labrum & Solomon, 2016; Ross & Coambs, 2018; Ross et al., 2022; Spivak et al., 2019). Mental illness also can impair one’s ability to work, which inevitably is associated with financial difficulties (Amin et al., 2023; Wigand et al., 2019).

Although the studies we found on psychological treatment for the financially stressed mostly were performed on chronically impoverished people, some reversable economic problems also can be prolonged. Food insecurity has been identified as a problem among college students, even at prestigious universities, where income might be expected to be above average later in life (Davis, 2015; Nam, 2023; Payne-Sturgis et al., 2018; Zein et al., 2019). Food insecurity has been identified among elite athletes training for Olympic games (Australian Broadcasting Corporation, 2023; Pells, 2020). The burden of educational debt among college graduates is currently a widely discussed issue in the United States (Koeze & Russell, 2022), and is associated with greater incidence of mental illness (Mac-Ginty et al., 2024; Webster & North, 2022; White, 2022). Debt from all sources has been linked to higher blood pressure and to poorer self-reported physical and mental health (Sweet, 2021).

The substantial proportion of Americans reporting financial stress in the American Psychological Association survey suggests that many people experience financial stress even when the objective criteria are not met. This discrepancy may be due to feelings of “relative deprivation,” i.e., perceived financial status compared to that of a reference group (e.g., neighbors or friends) (Park et al., 2017; Smith et al., 2012). Relative deprivation can impair feelings of well-being, increase disease susceptibility, and lead to a shorter life span (Chen, 2015), although it also can be associated with actual deprivation, including difficulty in accessing health care services (Boyle et al., 2004; Wilkinson & Pickett, 2007). In one study relative deprivation was not found to predispose people to cardiovascular illness, although the combination of low socioeconomic status (SES) and living in a neighborhood surrounded by other low SES households did (Boylan & Robert, 2017). Relative deprivation also has been found to be related to elevated incidence of anxiety, panic, and depression (Eibner et l, 2004; Nadler et al., 2020) and to poorer general physiological health (Park, 2021).

Psychological treatment, particularly with psychophysiological components such as relaxation training or biofeedback, can impact physiological as well as psychological symptoms associated with stress (Gevirtz, 2021). The stress response elicited by financial hardship does not necessarily differ physiologically from that elicited by other enduring stressors, so psychological methods for stress management should help the financially stressed population just as it helps other stressed groups, although presumably adding financial and/or job counseling to a treatment package may improve effectiveness.

Because of the association among stress, financial strain, and disease, it therefore is of interest to determine whether stress management methods can overcome the physiological effects of financial stress. This study reviews literature on psychological treatment of people with financial difficulties.

## Method

### Study Eligibility

Empirical studies were eligible for final inclusion if they met the following characteristics: (a) the targeted population included individuals who were financially stressed, (b) the study included at least one physiological outcome measure, and (c) a psychological intervention was used. We included published articles reporting intervention effects using randomized controlled trials, case–control studies, cohort studies, and similar designs. Only publications in English were assessed. We did not include books or chapters, as these outlets are not typically peer-reviewed, although we did include doctoral dissertations, which are usually subject to rigorous internal review. We only included studies having 10 or more participants.

### Search Strategy & Study Selection

A systematic literature search was conducted in *PsycInfo (EBSCO)* in May, 2023 including concepts of financial stress, psychophysiology, and intervention/treatment approaches. A total of 5071 articles were identified, of which 1957 were duplicates, resulting in 3114 articles for screening.

**Fig. 1:**
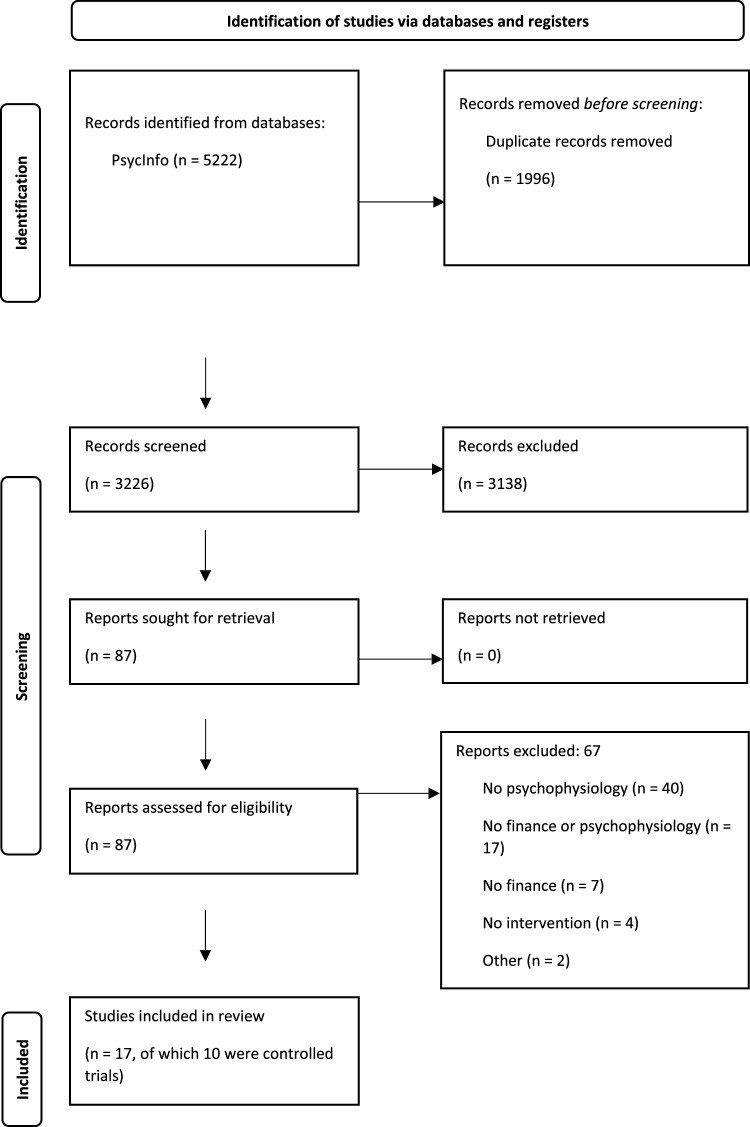
PRISMA flowchart

Article screening involved a two-level coding process. At the initial level of title/abstract screening, two reviewers (LD and JS) independently examined all articles (single coding process). Papers that were determined to be relevant for the review by either reviewer were designated for the second stage of the review, which involved a full-text evaluation. Eleven studies were agreed upon by reviewers and were independently evaluated by PL for final inclusion. See the PRISMA flowchart (Fig. 1) for the.

### Data Extraction

Data extraction and coding took place through Covidence (available at covidence.org). The following characteristics were extracted from each article: (a) population, (b) study design, (c) intervention design, (d) outcomes of interest (psychophysiological), (e) other modifiers such as date of publication, type of treatment, etc., and (f) study limitations as determined by coders and in the published reports. All members of the team completed the data extraction process. Data were scored on a spread sheet in a format that was compatible with the software used for meta-analysis.

### Meta-analysis

Following the initial review, we performed a meta-analysis on controlled studies with *n* ≥ 10 using Comprehensive Meta-Analysis (CMA), Version 4 (www.Meta-Analysis.com). A random effects model was used (see Borenstein, 2019; Borenstein et al., 2009) because studies were conducted using different populations, and Hedges’s *g* was used to assess effect size. When averaging across studies, the pooled effect size was weighted by the number of participants in each study. The *Q* statistic and a funnel plot were used to detect heterogeneity and outliers, and the I^2^ statistic (i.e., proportion of heterogeneity that is real rather than due to sampling error), with a criterion of 50%, was used to determine whether outliers could be interpreted. The prediction interval was computed to estimate with 95% certainty the range of *g* values within which results on any additional study with comparable methods on comparable populations might be expected to fall. Where identical procedures were conducted on separate populations in a single paper, we treated each population as a separate study unless combined data were presented in the published report. Where multiple time points were assessed in a study, we averaged the effect size across time points for the primary analysis. Although some studies examined ‘post-test’ and ‘follow-up’ assessments, it was not logical to do separate analyses of these points because some ‘follow-up’ periods were often shorter than some ‘post-test’ periods in other studies. Effect sizes were averaged across measures in the primary analysis when more than one physiological measure was used in a study, but we also looked at the effects on individual measures. A meta regression analysis was computed to assess the effect of time between pre-treatment and post-treatment evaluation, with each data point treated as an independent study. Effects at each time are displayed for each study. Effect sizes were computed only for controlled studies, although results of uncontrolled studies are also described in the systematic review, but not included in the meta-analysis.

## Results

A wide variety of concepts and measures of financial stress were used across the reviewed studies. Financial problems in the reviewed studies included personal or household finances (income, assets, debt, or other hardship) or unemployment or threat of it. These economic indicators were measured in various ways. Some studies measured the total amount of assets while others measured assets by counting the number of durable items owned by an individual or a household (such as refrigerators, microwaves, TV, cameras). The measures of debt were diverse, including the onset of debt, the amount of debt in general and of various types of debt, the debt-to-asset ratio, and debt problems like over-indebtedness, debt arrears, and perceived debt stress. Financial hardship was defined as difficulties in meeting the basic requirements of daily life due to a lack of financial resources. Examples include not having enough money for food, clothes, shelter, and medical expenses; being unable to pay bills on time or heat the home; having to sell assets; going without meals; or asking for financial help from others. Some studies examined the associations between depression and subjective perceptions of financial stress such as perceived financial hardship (e.g., subjective feelings of insufficiency regarding food, clothes, medical care, etc.), subjective financial situation (e.g., individual’s feelings of their overall financial situation), subjective financial stress, subjective financial position, financial dissatisfaction and so on. Some studies included risk of future financial hardship, such as probability of imminent layoff. None of the studies explicitly used criteria of relative deprivation. The psychological approaches to treatment included cognitive and behavioral interventions (e.g., cognitive behavior therapy, other forms of psychotherapy, social skills training, etc.), and psychophysiological interventions (e.g., relaxation, meditation, or biofeedback training).

### Systematic Review of Psychological Intervention Effects Among People with Financial Insecurity

#### Outcome Measures

Outcome measures in the selected studies included several physiological processes related to stress-related disorders and diseases. Heart rate variability was studied as a measure of general resilience (An et al., 2020; Perna et al., 2020). Greater amplitude of heart rate variability is related to more powerful function of various homeostatic systems. Each frequency of heart rate variability corresponds to a particular autonomic homeostatic reflex, some mediated by the sympathetic and others by the parasympathetic branch (Berntson et al., 1997). Diminished heart rate variability is related to a variety of diseases of the cardiovascular system (Milosky et al., 2022; Qiu et al., 2022), neural system (Heimrich et al., 2021; Siepmann et al., 2022a, 2022b; Stoco-Oliveira et al., 2022), reproductive system (Tiwari et al., 2022), renal (Shi et al., 2022), pulmonary system (Hogenson et al., 2021), gastrointestinal system (Yerushalmy-Feler et al., 2022), and various psychological and emotional systems (Benjamin et al., 2021; de Faria Cardoso et al., 2022; Lesnewich et al., 2019; Siepmann et al., 2022a, 2022b; Simon et al., 2022; Tonhajzerova et al., 2022). Indeed, decreased heart rate variability is associated with most physical and emotional disorders (Agorastos et al., 2023; Arakaki et al., 2023; D'Angelo et al., 2023; Kristal-Boneh et al., 1995; Siepmann et al., 2022a, 2022b). HRV values usually decrease during exposure to stress (Haque et al., 2024).

Inflammatory cytokines were studied because of the close association between stress and the inflammatory response (Kusnecov et al., 2021; Steptoe et al., 2007). Inflammation is the body’s preparation to defend itself from invasion, a prominent feature of the fight or flight reflex. Although protective against infectious disease, an excessive immune system activation is also associated with autoimmune and allergic reactions and presents a cardiac risk (Liu et al., 2017).

Corticosteroid output can be seen as homeostatic responses to inflammation (Barnes, 2006; McKay & Cidlowski, 2003). A meta-analysis by Adam et al. (2017) found that a flatter diurnal steroid decrease is associated with a variety of physical and mental diseases, including depression, cardiovascular disease, fatigue, and cancer (Adam et al., 2017). Interestingly, however, the opposite relationship has been found for anxiety, so increased cortisol is not universally related to the stress response. Sometimes cortisol output can be blocked in chronic stress, where homeostatic processes become dysregulated due to allostatic overload (Sze & Brunton, 2019). Some have speculated that corticosteroid output during stress may be related to the freezing response, which also is associated with reaction to severe uncontrollable stress and parasympathetically mediated stress symptoms (Korte, 2001). The freezing response is often associated with depression (Brierly and Jamieson, 1974) and trauma (Albanese et al., 2021) but anxiety is associated with greater activation (Brierly and Jamieson, 1974). Severe stress situations tend to elicit greater cortisol responses among individuals who freeze due to lack of ability to cope, while those trained to cope with stress show greater activation and a smaller cortisol reaction (Vit et al., 2023). Perhaps because of the complexity of homeostatic processes involved in steroid output, relaxation therapy does not have a consistent effect on it (Kische et al., 2022). Studies by Cohen et al., (1991, 1993) found increased disease susceptibility to rhinovirus infection among people exposed to stress, either induced acutely or through exposure to stressful life events. A probable link could be the association between steroids and immunosuppression in this study (Coutinho and Chapman, 2011).

Plasma adrenaline and noradrenaline also were studied as aspects of the sympathetic stress response, as described in more detail below. Higher blood pressure and obesity were studied as correlates of chronic stress and are prominent cardiovascular risk factors.

### Scoping Review of Psychological Treatment Among People with Financial Stress

In an uncontrolled study, Margolius et al. (2012) found significant decreases in blood pressure (mean of 21.8 mm Hg systolic pressure) over a six-month period in a low-income minority hypertensive population from a combination of home coaching and a program of regular home blood pressure monitoring. A separate group was additionally trained in home titration of blood pressure medication, but this add-on had no significant additional effect. Frequency of outpatient visits also decreased.

In a controlled study, Urizar and Muñoz (2011) studied the effects of a prenatal cognitive behavioral program among low-income Spanish speaking pregnant women with elevated risk for depression. Study participants were women with depression from a poor rural area, where income tended to be below accepted poverty lines. The program consisted of a 12-week prenatal course that focused on cognitive and behavioral stress management methods and helping women create a healthy physical, social, and psychological environment for themselves and their infants. At six months postpartum, the infants of people receiving the stress management program (*n* = 24) and infants of women with minimal risk for depression (*n* = 29) had a significantly lower cortisol level than those whose high-risk mothers received usual care (*n* = 33). Cortisol levels were lower among treated mothers at this time. At 18 months postpartum, women receiving the treatment also had significantly lower cortisol levels than those in the usual care group, but there were no between-group differences in cortisol among the children. Women in the treatment group reported more stress but less negative affect than women in the usual care group.

In another study from this group, Urizar et al. (2019) examined 88 pregnant women from a similar population, giving 55 of them a similar stress management program in an eight-week group format, with the rest receiving printed instructions about prenatal care. Of these, 62 were Latina and 26 non-Latina. Women with high prenatal anxiety in the treatment group showed a significantly smaller post-treatment decrease in diurnal cortisol levels than those in the control group. At three-month follow up, non-Latina women in the treatment group had significantly lower awakening cortisol levels than non-Latina women in the control group, but with a small effect size *g* = 0.262. Cortisol slopes were flatter in both groups during the second and third trimesters of pregnancy. They returned to baseline levels postpartum. There was greater flatness in the treatment group than in the control group during pregnancy, although with no significant differences in this measure for Latina women. This represented the opposite effect (i.e., relatively greater cortisol during the day) in the Treatment group compared with the Control group. The slope was steeper in the Treatment group postpartum, probably reflecting decreased cortisol during the day. There were no overall differences between the Treatment and Non-treatment groups during pregnancy averaging across ethnic groups and time periods. Only post-partum and in the third trimester did any cortisol measures correlate with perceived stress, suggesting that cortisol levels prior to that were part of the pregnancy process. For overall cortisol levels, there were no between-group differences either before or after pregnancy, either for Treatment vs. Non-treatment or for Latina vs. non-Latina comparisons. Non-Latinas had higher cortisol levels than Latinas during pregnancy. In the meta-analysis described below, time periods and ethnic groups were averaged. Women in the Treatment group reported significantly lower perceived stress levels than women in the Control group.

From the same investigative team, Miller et al. (2014) studied the effect of a seven-week family-oriented social intervention on reducing measures of inflammation. Treatment involved social skills and parental training, and instruction on sex and alcohol education. They studied 272 low-SES Black youth randomized to treatment and control groups. The control group consisted of printed material on child development and stress management. Intervention was given at the children’s age of 11, and outcome measures taken at their age of 19. Levels of inflammatory cytokines in a group receiving this intervention were lower at post-test than those among members of a control group who had not received this intervention. The effect size was large, *g* = 0.9. The investigators performed a mediation analysis showing that improved parenting (more nurturant-involved, less harsh-inconsistent) contributed significantly to the lower levels of inflammation in the intervention group. No self-report measures were reported for anxiety, stress, or mood.

Zhang and Emory (2015) conducted a randomized controlled trial, comparing an 8-session mindfulness intervention with a treatment as usual control condition among 65 pregnant financially stressed Black women. There were no significant differences between groups either in baseline cortisol levels or cortisol reactivity after exposure to a mild stressor (i.e., listening to a baby crying). On average, the stressor produced a decrease in cortisol level. In the treatment group, cortisol decreased significantly in response to the stressor with a large within-group effect size (eta^2^ = 2.9, p < 0.01), but there was no significant effect in the control group, with a small effect size *g* = 0.272. Between-group differences were not significant either for baseline cortisol or for the cortisol response. The intervention also decreased symptoms of stress and depression compared with the control condition, but none of these effects were apparent at a one-month follow-up.

Creswell et al. (2016) gave three days of mindfulness training at a relaxation retreat center to 18 unemployed adults reporting elevated levels of job-seeking stress. They compared effects to a randomly selected group of 17 individuals exposed to the usual relaxation experience of the retreat center. They measured functional connectivity in the default mode network (DMN), which measures goal-oriented thinking, often linked to depressive thoughts in clinical depression, and the left prefrontal cortex (LPFC), linked to executive function and modulation of emotional swings. They also measured interleukin-6 (IL-6), an inflammatory cytokine that has been linked to stress, and found a significantly larger decrease in the treatment group with a medium effect size, *g* = 0.57. They also found significantly greater increases in functional connectivity between the DMN and the LPFC. No verbal measures were reported of changes in stress, anxiety, or mood.

Toivanen et al. (1993) compared effects of a relaxation program, including muscle relaxation and relaxed five-second breathing, with an untreated group from the same population, on autonomic nervous system function among two groups of economically stressed people: low-level female bank employees with the prospect of imminent layoff (*n* = 48) and female hospital cleaning staff (*n* = 50). Both groups reported that they felt stressed. The intervention period was for six months. The bank employees received four 15 min sessions of training at the beginning of the period along with home training instructions while the hospital cleaners received three sessions per week. All groups were divided into treated and untreated conditions. The intervention consisted of muscle relaxation training and training in deep breathing at a rate of about six breaths/min. Treated groups showed a significantly greater tendency to approximate population values of heart rate variability after six months of treatment during a 6/min breathing test, *p* < 0.05, and a Valsalva maneuver, *p* < 0.001. Changes in heart rate variability did not differ between hospital cleaners and bank employees. We did not include this study in the meta-analysis because the study did not present data interpretable by Comprehensive Meta-Analysis, showing actual changes in heart rate variability in each group, and whether values increased or decreased. No verbal measures of stress, anxiety or mood were reported.

Toivanen et al. (1996) studied corticoid levels in these same economically stressed groups as well as a group of equally financially stressed low-income home health aides (*n* = 64). They analyzed the noradrenaline/adrenaline ratio, the cortisol/noradrenaline ratio, and the cortisol/adrenaline ratio. They interpreted all these values as indicative of greater stress. All decreased more in the treated group than in a no-treatment control group, although baseline differences between groups may have obscured some treatment effects. Greater decreases in the questionnaire measures of stress at three months tended to correlate with decreases in cortisol and noradrenaline. Although measures of stress tended to decrease at three months, they appeared to rebound at six months, but the rebound was smaller in the treated group.

Samuel-Hodge et al. (2009) gave a behavioral weight loss program to 72 low-income women while 71 women from the same population were randomly assigned to a no-treatment control condition. While obesity is not necessarily a stress symptom, it is often a particular problem for low-income women (Griffith, 2022) and is common among people exposed to chronic stress (Tomiyama, 2019). The treatment consisted of 16 weekly group sessions while women in the control group received two newsletters. Assessed after five months, women in the treatment group lost significantly more weight and had significantly greater decreases in blood pressure and cholesterol levels than women in the control condition. There were no between-group differences in measures of depression or quality of life. Because weight loss counseling is qualitatively different from psychotherapy or stress management therapies, we did not include this study in the meta-analysis described below.

In a doctoral dissertation, Brown (2016) reported results of a six-session weekly mindfulness meditation program adapted for child welfare families with substance abuse (MORE-CW). Participants (*n* = 15 experimental, *n* = 13 wait list control) were assessed for changes in heart rate variability, parental stress, coping, and mindfulness as well as parental substance misuse and child maltreatment. Significant differences were found between groups, such that MORE-CW was associated with reduced self-reported parenting stress, child abuse potential, child behavior problems, and improved mindfulness. Significant group by time differences were found on participant heart rate variability from pre- to post-assessment, with greater increases occurring in the treatment group.

Lei et al., (2022a, 2022b) used data from the Protecting Strong African American Families (ProSAAF) project, a randomized controlled trial (*n* = 348) of a written intervention designed to improve marital communication, compared with an untreated control group. The aims of this study were to evaluate the effect of treatment on the accelerated financial-strain-related aging effect. Their measure of aging, the, DunedinPoA (Dunedin[P]ace[o]f[A]ging), was an “index of 28 biomarkers related to cardiovascular, metabolic, pulmonary, renal, hepatic, immune, and dental health systems” devised by Belsky et al. (2020). They studied African Americans from a low-income neighborhood, mean age = 36.1 years. Although 33% of the population had incomes at or above 150% of the federal poverty level, we included this study because the great majority of study participants were poor, with median monthly income of $1,375. There was a significant effect of cumulative financial strain on accelerated pace of aging after controlling for covariates and cell compositions (*β* = 0.10, *p* = 0.03; Cohen’s *d* = 0.32) with a medium to large effect size. The ProSAAF intervention led to improvement in couple functioning from baseline to a 24-month follow-up (r = 0.15, p < 0.01). Treatment related changes in couple function had a buffer effect on these findings such that poorer functioning showed accelerated aging, while better functioning diminished the effect of financial strain on aging. Treatment-related improvement in couple functioning also was significantly more improved in the treatment than control group and had a buffering effect on the relationship between financial strain and aging, such that better marital functioning diminished the effect of financial strain on aging.

Wadsworth et al. (2022) studied the effect of a comprehensive (32 h) group-based program aimed at enhancing emotional coping strategies. The intervention program targeted proximal mechanisms of stress adaptation in early adolescence to prevent mental health problems in youth living in poverty. The study focused on understanding the impact of the intervention on psychophysiology and poverty-related stress. Participants were a sample of youth (*n* = 113) aged 11 to 14 years from low-income backgrounds who were randomly assigned either to the intervention group or to a no-treatment control group. Other outcomes included measures of stress coping, assessed by a combination of written parent and youth reports as well as via interviews and questionnaires. Cortisol was measured several times during each session, which included exposure to the Trier test of social stress. Significant decreases in peak cortisol levels during the session and improvement in stress coping were found after the intervention and were maintained at a 12-month follow-up period, with a small to medium effect size.

In an intervention directed at moderating the quality-of-life impact of poverty, Lloyd et al. (2008) assessed the effect of improving thermal quality of housing to eliminate damp and moldy living environments on blood pressure (BP), general health, and financial status among individuals living in “rehoused” flats from the “inner city slums in Glasgow,” United Kingdom. Participants (*n* = 75 active arm;* n* = 40 control arm) were visited in their homes to record blood pressure (BP) before the residents of the intervention group flats were upgraded and one year after living in their upgraded flat. Control group assessments were taken at the same time. At baseline, there were no differences in initial BP readings nor between men and women in the same age group. In the intervention group there was a significant decrease in systolic (*p* < 0.001) and diastolic (*p* < 0.001) BP. In the control arm where there was a small, non-significant decrease in systolic pressure and a slight increase in diastolic pressure (*p* < 0.011). Residents self-reported changes in finances by noting that heating costs had significantly decreased (along with warm water costs) and that there was no increase in rent. No observed changes were made in the control arm. Although stress reduction may have been an intervening variable between apartment improvement and blood pressure, this connection was not clear, and the intervention was not a psychological technique, so we omitted this study from the meta-analysis described below.

### Effect of Financial Counseling on Psychological Adjustment

Presumably obtaining more money would directly contribute to people facing financial stress, even if it does not, by itself, necessarily contribute to managing other kinds of stress. Financial or employment counseling to this end may be an appropriate avenue for investigation, but we found no studies that examine the effects of these interventions on physiological indicators of stress. One study found that financial counseling helps people improve their financial knowledge and coping, but with little effect on verbal measures of financially related stress. An intervention to improve quality of housing, which may be considered a financial intervention, improved physiological stress effects (Lloyd et al., 2008).

Ford et al. (2017) assessed palmar skin conductance among couples during financial discussions and found that those with higher arousal levels were more likely to indicate that they would seek help of a professional financial planner. In an uncontrolled demonstration project, Britt-Lutter et al. (2019) gave 13 couples a five-session course over a five-to-six-week period to guide conversations about how money affects their relationship. It included elements of training in communication skills, stress management, and financial management. Participants exhibited significant decreases in relationship and financial stress both post-treatment and at a three-month follow-up, but no change in hand temperature, which was high, in a relaxed direction, at the beginning of treatment. Pre-treatment hand temperature predicted post-treatment financial and relationship stress.

### Meta-analysis of Physiological Effects of Stress Management Methods in Financially Stressed Individuals.

Of the 16 papers accepted for this review, nine contained data adaptable for analysis using Comprehensive Meta-Analysis. Of these, one described data from two populations, each analyzed as a separate study. Characteristics and findings of these studies are summarized in Table 1. In this analysis we only included controlled studies of a psychological treatment among financially stressed individuals where physiological outcome measures were used. We found no studies meeting these criteria that included a financial counseling component.

**Table 1:** Studies Included in Meta-Analysis: Individual Measures, Subgroups, and Time Points

Study	Population	Intervention	Measure	Effect size (*g*)	*# visits*	*# Months*
Brown (2016)	Child welfare parents (*n* = 21)	Mindfulness training	Heart rate variability (RMSSD)	1.26	6	1.5
Cresswell et al. (2016)	Unemployed adults (*n* = 35)	Mindfulness training	IL-6	0.557	4	1.0
Lei et al., (2022a, 2022b)	Low-income Southern Black Americans (*n* = 499)	Relationship enhancement training	DNA assessment of aging	0.322	8	9
Miller et al. (2014)	Low SES Black children (*n* = 272)	Parenting training for pregnant mothers	Inflammatory cytokines	0.901	7	96
Samuel-Hodge et al. (2009)	Obese low-income adults (*n* = 127)	Dietary Instructions	% body fat Disastolic BP	0.541 0.267	16	5
			HDL-C	0.250		
			Systolic BP	0.603		
			Weight	1.377		
Toivanen et al. (1996)	Hospital cleaning	Relaxation	Cort./Adren. Ratio	0.021	36	6
	Staff (*n* = 82)	Training	Cort./Nor. Ratio	0.248		
			Nor. / Adren. Ratio	0.000		
	Home health aides		Cort/Adren Ratio	0.134		
	(*n* = 43)		Cort/Nor ratio	0.418		
			Nor/ Adren Ratio	0.077		
	Bank employees		Cort./Adren. Ratio	− 0.254		
	Facing		Cort./Nor. Ratio	− 0.087		
	Unemployment		Nor. / Adren. Ratio	0.721		
	(*n* = 48, 6 laid off)					
Urizar and Muñoz (2011)	Infants of low-income mothers (*n* = 46)	Relaxation training for mothers	AM Cortisol 6 months^a^	0.312	1	18
			PM Cortisol 6 months	0.246	2	
			AM Cortisol 18 months	0.427		
			PM Cortisol 18 months	0.074		
	Low-income mothers (*n* = 53)	Relaxation training	AM Cortisol 6 months^b^	0.635		
			PM Cortisol 6 months	0.197		
			AM Cortisol 18 months	0.246 0.143		
			PM Cortisol 18 months			
Urizar et al. (2019)	Low-income pregnant women (*n* = 100)	Stress management	Diurnal cortisone		8	
			slope^c^	− 1.731		3
			2nd trimester	− 1.757		6
			3rd trimester	0.586		18
			postpartum			
Wadsworth et al. (2022)	Low-income early adolescents(*n* = 129)	Stress coping training	Peak cortisol during testing session	0.379	16	12
Zhang and Emory (2015)(*n* = 30)	Low SES Black women	Mindfulness training	Baseline cortisol	0.194	4	1
			Cortisol response	0.272		

*SES* Socioeconomic status

Unless noted otherwise, effect sizes were calculated on differences in changes from baseline in treatment and control groups

^a^Age of infant. Mothers were trained during pregnancy. Treatment versus control mean at each time period

^b^Weeks postpartum. Treatment vs. control change from pregnancy in each time period

^c^Change from pretest given before training in the first trimester

**Fig. 2:**
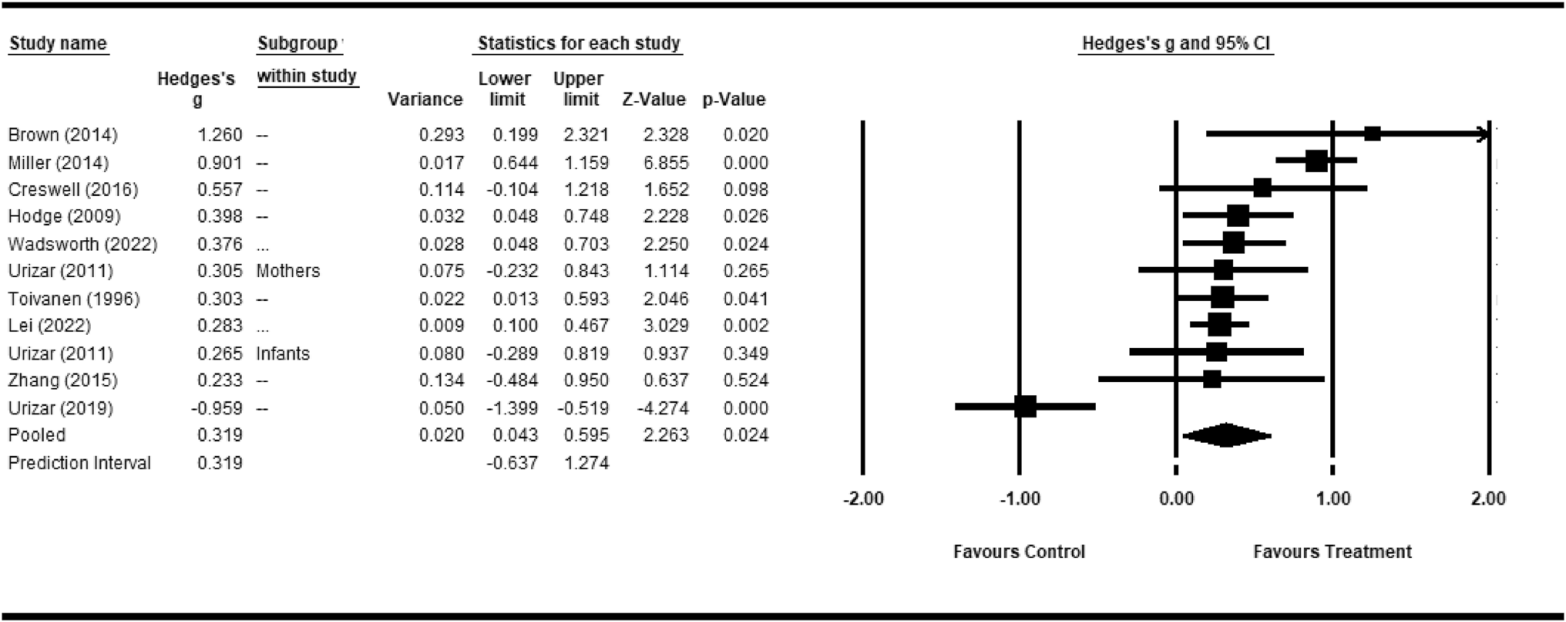
Forest plot of studies in meta-analysis. *g* = 0.448 *p* < 0.001 with Urizar et al. (2019) excluded

**Fig. 3:**
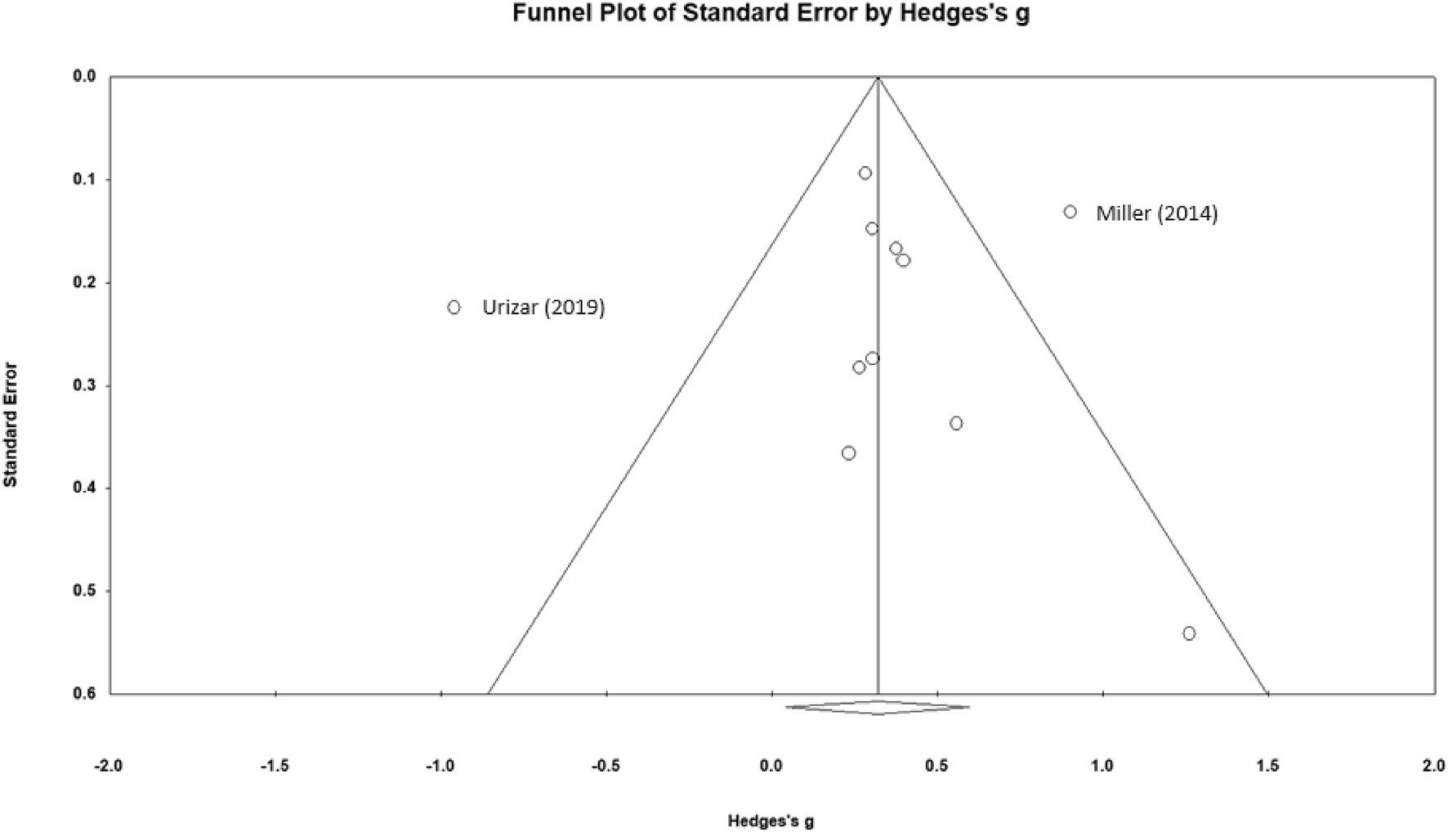
Funnel plot of Hedges’ *g* distribution, all studies, averaged across measures and time points

Figure 2 shows a forest plot of Hedges’ *g* values. The pooled weighted values of *g* show a small to medium effect size, *g* = 0.319, *p* < 0.03, with a 95% prediction interval between a medium negative effect, *g* = −0.637 and a very large effect, *g* = 1.363. There was significant heterogeneity, *Q* = 55.749, *p* < 0.001, which is considered interpretable, with larger between-study than within-study variance, *I*^*2*^ = 82.062% (Table 2). Figure 3 is a funnel plot showing two outlying studies, one with positive results and one negative. Of these, the larger negative outlier is Urizar et al. (2019), entirely explained by the flatter diurnal cortisol response during pregnancy, although this was reversed to a medium positive effect size, *g* = 0.586, postpartum. Without the anomalous pregnancy data, the effect size was medium, *g* = 0.457, *p* < 0.001, but still with significant heterogeneity*, Q* = 20.021, *p* < 0.03. When eliminating both Uzair et al. (2019) and the positive outlier (i.e., Miller et al., 2014), the effect size was small to medium, *g* = 0.333, and still significant *p* < 0.001. In this analysis there were no outliers, and the heterogeneity was not significant, *Q* = 4.134, *p* = 0.845. With a variance of 0.004, the prediction interval was incalculably small, with little variance around *g* = 0.333 (Table 3).

**Table 2:** Meta-analysis statistics all studies, averaged across time points and measures

Model	Effect size and 95% confidence interval											Test of null [2-Tail]				Prediction interval		
Model	Number studies	Point estimate		Standard error		Variance	Lower limit			Upper limit		Z-value		P-value		Lower limit		Upper limit
Fixed	11	0.353		0.053		0.003	0.248			0.457		6.601		0.000				
Random	11	0.319		0.141		0.020	0.043			0.595		2.263		0.024		− 0.637		1.274

Between study		Other heterogeneity statistics			
Tau	TauSq	Q-value	df [Q]	P-value	I-squared
		55.749	10	0.000	82.062
0.398	0.159

**Table 3:** Meta-analysis statistics without Uzair et al. (2019) and Miller et al. (2014), averaged across time points and measures

Model	Effect size and 95% confidence interval											Test of null [2-Tail]				Prediction interval		
Model	Number studies	Point estimate		Standard error		Variance	Lower limit			Upper limit		Z-value		P-value		Lower limit		Upper limit
Fixed	9	0.332		0.061		0.004	0.213			0.451		5.479		0.000				
Random	9	0.332		0.061		0.004	0.213			0.451		5.479		0.000				

Between study		Other heterogeneity statistics			
Tau	TauSq	Q-value	df [Q]	P-value	I-squared
		4.038	8	0.854	0.000
0.000	0.000				

Note that the two outlying studies had unusual characteristics. Urizar et al. (2019) was the only study to measure steroids during pregnancy. Pregnancy has a profound effect on steroid output. The study by Miller et al. (2014) was one of two studies using inflammatory cytokines as an outcome measure, yielding a large effect size, *g* = 0.914 with a large participant pool, *n* = 272. A smaller study on an inflammatory cytokine, *n* = 35, found a medium effect size, *g* = 0.570. Another smaller study (Brown, 2016) (*n* = 21) found an even larger effect size, *g* = 1.26, *p* < 0.02 for heart rate variability. Because of the small study size, however, the effect size in that study was within the expected range of heterogeneity because of the possibility of the small sample size (Fig. 3).

Pooled effect sizes were small to medium for cortisol measures, *k* = 6, g = 0.396 across six studies, not including pregnancy data in Urizar et al. (2019). Larger effect sizes were found for the combination of HRV and inflammation, which had a large, pooled effect size (*k* = 3,* g* = 0.876). The difference was significant, *Z* = 7.876, *p* < 0.001. A meta regression analysis was performed for pre-post time interval, without the outlying positive study with a long (96 month) pre-post period and a high effect size (Miller et al., 2014) and both with and without another outlying study by Brown (2016) with a short (1.5-month pre-post period). Measures taken during pregnancy in the Urizar and Muñoz (2011) study were excluded. The regression coefficient for pre-post duration on effect size was 0.0004 and nonsignificant, *Q* = 0.000, *p* > 0.9 without either outlier. With Brown (2016) included, the coefficient is still nonsignificant -0,003, *Q* = 0.06, *p* = 0.8102.

## Discussion

Despite the heterogeneity of psychophysiological measures, treatment procedures, and populations in the reviewed studies, there is convincing evidence that, despite the continuing nature of financial stress in the populations studied, psychological stress management and various other behavioral interventions targeted at general psychological adjustment have significant beneficial psychophysiological effects on financially stressed populations. All the studies included a stress management component. Some included training in behavioral skills but none of the studies included in the meta-analysis had an element of financial counseling. Although the effects on general health have yet to be examined, significant findings for blood pressure, heart rate variability, and inflammatory cytokines, all of which are related to a variety of serious diseases, suggest that, in the long term, the health benefits could be considerable. In general, it appears that the various stress management methods reviewed here appear to have had remarkably similar results to these same methods in other populations, not necessarily financially stressed (Lehrer & Woolfolk, 2021). This review shows that the distractions caused by financial stress, often chronic, does not impede the effectiveness of these methods.

This review also highlights some important gaps in the literature. It is surprising that interventions targeting financially stressed groups mostly failed to directly address financial remedies for financial problems. Although most people, not just those experiencing financial stress, might benefit from counseling about financial planning, the financially stressed might benefit particularly. Financial counseling has been integrated with other forms of psychotherapy in the new discipline of ‘financial therapy’ (Klontz et al., 2015, 2016). Indeed, financial counseling has long been identified as a needed component in family therapy (Klontz et al., 2015; Myhre & Sporakowski, 1986). In financially stressed groups, financial counseling may directly contribute to reducing experience of stress.

There is some evidence from more cursory studies that financial counseling might help with stress, although there are no physiological data. A study of solution-focused financial therapy targeting financial goal planning among students in a financial planning course found a reduction in financial anxiety (Archuleta et al., 2020), and another study found that financial counseling (without added psychotherapy) reduced stress symptoms associated with financial problems (Britt & Tibbetts, 2015). A third study showed that the combination of financial counseling and marital therapy can improve marital happiness (Falconier, 2015). A fourth study, however, found that financial counseling alone is not sufficient to reduce levels of general stress. Research is needed to determine whether adding financial counseling to standard stress management methods strengthens the physiological effects of therapy. An obvious experimental design would be to compare stress management alone with the same treatment but including an element of financial planning.

### Sensitivity of Outcome Measures

Inflammatory cytokines may be particularly sensitive to stress management methods, as found in the outlying study by Miller et al. (2014). The effect of decreases in inflammation on psychophysiological symptoms was not studied but could be considerable because many psychiatric symptoms (Franco et al., 2017; Lee, 2020) and somatic symptoms (Almulla et al., 2023; Kim et al., 2023), sometimes mediated by depression Jayakumar et al. (2023), are associated with an inflammatory response.

Another particularly sensitive measure may be heart rate variability, which is a measure of resilience and health, but this measure was used in only one study. Heart rate variability biofeedback has major effects on a variety of stress related symptoms (Lehrer et al., 2020), but this intervention was not used in any of the reviewed studies. Training in this method can now effectively be delivered remotely (Economides et al., 2020), using inexpensive wearable digital devices or cell phone applications (apps).

The inconsistent effects for cortisol measures may reflect the homeostatic nature of the cortisol response. If the major stress effect is inflammatory, as part of the immune system’s preparation to protect the body from effects of possible injury, then the corticosteroid response, which is anti-inflammatory, may be seen as a homeostatic response (Hamilton et al., 2024; Roy et al., 2024). It is heightened during certain stages of pregnancy and may not yet be mobilized in response to acute stressors. Measures of inflammation and cortisol were not taken at comparable time periods in the studies we reviewed, so the dynamics of this interaction cannot be demonstrated in the studies reviewed here.

### Limitations and Need for Future Research

All the studies except two, the subgroups of bank employees facing the possibility of unemployment (Toivanen et al., 1993, 1996), examined populations with chronic poverty. We found no studies of other financially stressed populations, such as the transitionally financially stressed (e.g., elite athletes, college students, etc.) or people with some financial means who experienced relative deprivation. However, the focus on financially stressed populations highlights the absence of financial counseling as a component in therapy. The fact that most populations in this study were chronically poor reinforces this observation. Poor populations tend to have lower levels of financial literacy (Chien & Karlson, 2018) and less knowledge and resources to obtain it. Financial counseling could be of obvious benefit.

Additionally, research is needed on the role of psychological treatment in preventing or ameliorating mental or somatic diseases for which the physiological measures reported here are indicators.

Notably, the studies reviewed in this study did not include the use of newer digital technologies, such as on-line physiological monitoring and biofeedback and remote treatment, which can increase accessibility of treatment (Economides et al., 2020). The finding of particularly strong therapy effects on heart rate variability suggests that heart rate variability biofeedback might be tried as an intervention strategy that can easily be done remotely using such easily available telephone apps such as *Elite HRV¸ HRV4Biofeedback*, and *Inner Balance* (Birk et al., 2024; Hirten et al., 2024)*.*

Few of the studies included adequate attention-placebo controls. These could include such procedures as biofeedback training with neither arousing nor relaxing effects as used by studies from Mather’s laboratory (Nashiro et al., 2023), or psychoeducational lectures on the nature of stress. Such a control would show that the intervention had a specific stress-reducing effect, separately from the effects of attention from an expert on stress reduction.

All studies relied on self-report of protocol adherence as a measure of actual adherence. Self-reports often produce inflated estimates (Stiratt et al., 2015). Additionally, effects of medications were not controlled in most studies, and effects on particular physiological outcomes were each assessed in very few studies, so replication is needed.

## Conclusions

Studies combining various psychological approaches to stress management show that these methods have a beneficial physiological effect on financially stressed people. Evidence for this conclusion was found among mostly chronically poor populations, despite the continuing nature of this stress. Treatment-produced decreases in inflammatory cytokines and increases in heart rate variability may be the most robust physiological effects, although additional studies are needed to verify this. More research is needed to evaluate the relative effects of various outcome measures and therapy components, including the inclusion of financial therapy to the treatment package. The data reviewed here lay a groundwork for the nascent field of financial psychophysiology. This review outlines important research needs in this field.
